# Role of Individual Clinician Authority in the Implementation of Informatics Tools for Population-Based Medication Management: Qualitative Semistructured Interview Study

**DOI:** 10.2196/49025

**Published:** 2023-10-24

**Authors:** Allison Ranusch, Ying-Jen Lin, Michael P Dorsch, Arthur L Allen, Patrick Spoutz, F Jacob Seagull, Jeremy B Sussman, Geoffrey D Barnes

**Affiliations:** 1 Center for Clinical Management Research Veterans Affairs Ann Arbor Healthcare System Ann Arbor, MI United States; 2 Center for Bioethics and Social Sciences in Medicine University of Michigan Ann Arbor, MI United States; 3 College of Pharmacy University of Michigan Ann Arbor, MI United States; 4 Institute for Health Policy and Innovation University of Michigan Ann Arbor, MI United States; 5 Veterans Affairs Salt Lake City Health Care System Salt Lake City, UT United States; 6 Veterans Integrated Service Network 20 Northwest Network Vancouver, WA United States; 7 Department of Internal Medicine University of Michigan Ann Arbor, MI United States; 8 Division of Cardiovascular Medicine, Department of Internal Medicine Frankel Cardiovascular Center, University of Michigan Ann Arbor, MI United States

**Keywords:** direct oral anticoagulant, population management, implementation science, medical informatics, individual clinician authority, electronic health record, health records, EHR, EHRs, implementation, clotting, clot, clots, anticoagulant, anticoagulants, dashboard, DOAC, satisfaction, interview, interviews, pharmacist, pharmacy, pharmacology, medication, prescribe, prescribing

## Abstract

**Background:**

Direct oral anticoagulant (DOAC) medications are frequently associated with inappropriate prescribing and adverse events. To improve the safe use of DOACs, health systems are implementing population health tools within their electronic health record (EHR). While EHR informatics tools can help increase awareness of inappropriate prescribing of medications, a lack of empowerment (or insufficient empowerment) of nonphysicians to implement change is a key barrier.

**Objective:**

This study examined how the individual authority of clinical pharmacists and anticoagulation nurses is impacted by and changes the implementation success of an EHR DOAC Dashboard for safe DOAC medication prescribing.

**Methods:**

We conducted semistructured interviews with pharmacists and nurses following the implementation of the EHR DOAC Dashboard at 3 clinical sites. Interview transcripts were coded according to the key determinants of implementation success. The intersections between individual clinician authority and other determinants were examined to identify themes.

**Results:**

A high level of individual clinician authority was associated with high levels of key facilitators for effective use of the DOAC Dashboard (communication, staffing and work schedule, job satisfaction, and EHR integration). Conversely, a lack of individual authority was often associated with key barriers to effective DOAC Dashboard use. Positive individual authority was sometimes present with a negative example of another determinant, but no evidence was found of individual authority co-occurring with a positive instance of another determinant.

**Conclusions:**

Increased individual clinician authority is a necessary antecedent to the effective implementation of an EHR DOAC Population Management Dashboard and positively affects other aspects of implementation.

**International Registered Report Identifier (IRRID):**

RR2-10.1186/s13012-020-01044-5

## Introduction

With growing use since their introduction in 2010, direct oral anticoagulants (DOACs) are now the most commonly prescribed anticoagulants to prevent stroke in patients with atrial fibrillation and to prevent or treat venous thromboembolism. Despite their high degree of efficacy, DOACs remain high-risk medications that can cause severe and fatal complications when prescribed inappropriately [1,2]. Given that inappropriate DOAC prescribing occurs in up to one-quarter of patients, health systems are implementing population health tools that leverage the power of clinical data in electronic health records (EHRs) to evaluate DOAC-prescribing trends and act as a clinical decision support (CDS) informatics tool for identifying patients with potential medication errors. One good example is the DOAC Population Management Tool (or “DOAC Dashboard”) developed by the Veterans Health Administration (VA) and implemented in the nationwide VA health system [3,4]. As the data necessary to determine appropriate DOAC prescribing is contained within the EHR, the VA’s DOAC Dashboard is an effective CDS tool used for advancing anticoagulation stewardship [5,6], modeled after successful antimicrobial stewardship efforts [7].

EHR-based informatics tools, such as the DOAC Dashboard, allow for efficient oversight and management of large patient populations. However, implementing these EHR-based tools and empowering staff to use them for patient benefit remains a challenge, with many barriers and facilitators to their adoption [8]. The empowerment of nonphysician staff with medication management expertise and available time is a significant concern, as the nonphysician staff may face limitations in their authority to manage medication due to organizational or legal rules. The success or failure of implementing EHR-based tools may hinge upon barriers and facilitators such as the level of individual authority given to clinicians [8-10], which is also a key factor for effective interprofessional collaboration [11,12].

The term authority has been used to encompass many related concepts. The most formal definition of authority refers to the power granted to individuals to carry out role-related functions. Such authority is legitimized through consensual agreements codified in laws, organizational policies, contracts, and other accepted institutional frameworks [12,13]. In the clinical domain, a clinician’s authority to prescribe medication or provide care depends on authority granted by the institution or licensure body. In the specific context of this study conducted in the State of Michigan, it is noteworthy that clinical pharmacists and nurses lack the legal authority to prescribe DOAC medications. Consequently, they depend on physicians and other clinicians who possess prescribing authority.

Within the context of organizational behavior, authority can be seen as a dynamic concept that emerges from interactions between individuals negotiating the scope of power they have over one another and their tasks [14]. This definition holds particular significance when examining the interactions between prescribing clinicians and anticoagulation pharmacists and nurses as it directly impacts the implementation of a population health management tool for anticoagulation stewardship.

Authority’s influence impacts related concepts of autonomy, such as control over one’s own work (scheduling, staffing, and workflow), control over the flow of information (communication), and control over the implementation of and use of technology. Providing nonphysicians with authority over workflow and staffing can improve their job satisfaction, while the lack of such authority can be detrimental [15-17]. Relating to communication, authority structures, such as power distance [18,19], also have an impact on both EHR implementation and patient safety. For example, when a strong hierarchical authority dynamic exists between medical doctors and nonphysician professionals, it can result in impediments to effective communication and sound clinical decision-making [20,21]. Granting frontline clinicians the authority to optimize EHR can also have a positive impact on both job satisfaction and patient safety, while the lack of such authority may lead to negative consequences [22-24].

In previous work on the topic of dashboard implementation [8], the perceptions of dashboard success were closely tied to issues related to authority. Our team examined the perceived barriers to implementation success in the VA health system after the dashboard implementation and in non-VA sites before it had been implemented. Through extensive interviews of users within the VA sites and non-VA sites, five key determinants of implementation success emerged: (1) clinician authority and autonomy; (2) communication, documentation, and administrative needs; (3) staffing and work schedule; (4) integration with existing information systems; and (5) clinician self-identity and job satisfaction. One of the key differences between the non-VA setting and the VA setting was concerns about authority and autonomy. The VA sites had higher baseline levels of authority and autonomy and voiced more concern about the Dashboard implementation, and the non-VA sites had lower baseline levels of authority and autonomy and voiced less concern.

This difference may be related to the level of authority that the 2 systems grant their nurses and pharmacists. Individual clinician authority, especially for nonphysicians, can vary significantly from health system to health system. This is particularly true in non-VA health systems, where pharmacists and nurses do not have as much legal or organizational authority over clinical and operational roles. Non-VA nurses and pharmacists are required to operate under individual state rules and regulations as well as often working with independent, self-employed physician groups. In addition, differences between the 2 systems in information flow also were cited. Non-VA nurses and pharmacists cited concerns about a lack of access to medical records from outside their health system, a barrier not frequently noted by VA interviewees due to the availability of nationwide VA EHR records.

A limitation of our previous research was that the DOAC Dashboard had not yet been implemented outside of the VA system, and our ability to draw conclusions about barriers and facilitators to implementation was relegated to government health care systems. Examining the influence of authority and related concepts on the implementation of the DOAC Dashboard in non-VA settings could expand our understanding. As an increasing number of health systems look to expand the use of EHR-based tools for population-level patient management, addressing issues surrounding authority may be critical for achieving success.

This study aims to gain a better understanding of the role and influence of authority and related concepts on the implementation of EHR-based tools. This understanding is critical for the broad adoption of this specific EHR-based tool and for future implementation efforts for EHR-based clinical and population-level tools. Using the DOAC Dashboard and safe DOAC prescribing as an exemplar, this study will focus on the following questions: (1) whether and how the use of a DOAC Dashboard empowers the individual authority of pharmacists and nurses to ensure the safe use of DOACs and (2) how the implementation and adoption process create or harm individual authority in ways that facilitate or hinder the use of a DOAC dashboard (eg, regulatory, resource, and interprofessional communication).

## Methods

### Setting and Participants

We conducted semistructured interviews with anticoagulation professionals working in 3 regional health systems, all of whom had implemented an EHR-based population health management tool for DOACs, the “DOAC Dashboard,” within their Epic EHR system (Epic Systems Corporation) [8]. These sites had all previously participated in the interviews that were conducted before the implementation of the DOAC Dashboard in their health systems. Clinicians at these sites were approached via email following the implementation of the DOAC Dashboard for a second round of interviews.

The participants interviewed were a purposeful sample of clinical pharmacists and nurses involved in patient monitoring and care in anticoagulation clinics. As this study was a follow-up to our previous investigation, we were limited to the sample of non-VA institutions that had implemented this dashboard. There are only 4 institutions that have implemented this dashboard, and within each institution, only a limited number of individuals work with the dashboard. Although a small absolute number, our sample includes a large proportion of all individuals working with the dashboard. These individuals’ experiences using the dashboard reflect the commonality and diversity in the implementation of this population management tool across health systems. Some of these participants may have participated in preimplementation interviews included in the previous data set; however, as all our interview data were deidentified, participation could not be tracked between data sets.

### Ethical Considerations

All participants provided verbal consent for participation and recording, and each transcription was deidentified, following an institutional review board–approved protocol. This project was reviewed and approved by the institutional review board at the University of Michigan (HUM00162234).

### Data Collection

Semistructured interviews with a focus on clinicians’ empowerment over their workflow, as well as their work on and within the DOAC Dashboard, took place from August to September 2022. Our semistructured interview guide was developed and pilot-tested to ensure the clarity of the questions and prompts. Interviews were conducted by a primary and a secondary interviewer (AR and YJL), who are both trained and have previous experience in conducting semistructured interviews with health care professionals. Both interviewers are female qualitative analysts.

The interviews were conducted via Zoom (Zoom Technologies), with only the research team and the interviewee present during the interview. Each interviewee was interviewed once during this process. The interviews were audio recorded, and transcripts were created via the recording and transcription functions on Zoom. The secondary interviewer also took detailed notes of the interviews. We did not return transcripts to participants for comments or clarification. The interview team verified the transcription by comparing the transcription to the audio and made any necessary corrections. The team also edited for clarity to concisely convey the participants’ message (eg, removal of “ums” and “uhs”) and deidentified the transcripts.

### Qualitative Analysis

The research team used the method of content analysis to analyze their data. The transcripts were coded by 3 team members (AR, YL, and FJS) for the five key determinants of implementation success from our previous research [8]: (1) clinician authority and autonomy; (2) communication, documentation, and administrative needs; (3) work scheduling and staffing; (4) integration with existing information systems; and (5) clinician self-identity and job satisfaction. Expanded definitions of each determinant are included in the attached codebook (Multimedia Appendix 1). We changed the label of “clinician authority and autonomy” to “individual clinician authority” to better reflect the themes that emerged from the interviews. As noted in the introduction, the theme of authority comprises many legal, organizational, and interprofessional concepts. When coding individual clinician authority, we maintained a single code to reflect the integration of various aspects of individual authority and recognized that related but discrete concepts may also be present in any given statement or segment.

Before coding the transcripts, the application of the 5 codes was discussed until a consensus was reached. We noted that the job satisfaction code from the VA transcripts focused on a concern that the respondents had about being “replaced by the Dashboard.” However, the code for job satisfaction in current interviews included additional and more general job satisfaction issues that emerged as a theme in the current interviews.

Using Excel (Microsoft Corp), we parsed the transcripts into interview segments consisting of an answer to a main interview question, representing a complete thought. Segments may contain follow-up or clarification questions by interviewers. Segments may also include only a portion of an answer to an interviewer if the complete answer contained 2 or more concepts. Each transcript was reviewed and coded by 3 team members (AR, YJL, and FJS) independently for the 5 determinants of implementation success. Transcripts were reviewed by the team, and discrepant codes were reconciled through discussion and consensus.

Each segment was coded for any applicable determinants present, so a single segment may be coded as containing multiple relevant determinants. Each coded segment was also scored by consensus as containing sentiments that reflected positively regarding the presence of the determinant or reflected negatively regarding the absence of the determinant. For example, a statement such as “we are able to…” or “we have the flexibility to…” may be considered a positive example of the determinant. On the other hand, a statement like “we have no control…” or “we aren’t able to…” may be considered a negative example of the determinant.

To better understand the subcomponents of individual clinician authority, we examined the co-occurrence of that determinant with the other 4 determinants. Each segment had been coded independently for any of the 5 applicable determinants. We aggregated segments that contained both individual clinical authority and one other determinant and reviewed the aggregated segments for thematic patterns.

## Results

### Overview

We conducted interviews at all 3 non-VA sites, and our study included participants who worked closely with the DOAC Dashboard. This resulted in 6 interviews, with 3 anticoagulation nurses and 3 anticoagulation pharmacists being interviewed individually via video conference. All worked at 1 of the 3 non-VA sites using a DOAC Dashboard. The average interview length was 28 (range 24-36) minutes.

In order to gain insight into the ways that the use of the DOAC Dashboard may empower individual authority (our first research question), we examined the themes brought forth by the interview participants. Therefore, each interview was parsed into segments reflecting a thematic unit. These segments were each coded into 1 or more of the 5 determinants as described above. This resulted in 108 separate segments. Code frequencies within the 108 segments are shown in Table 1.

**Table 1:** Determinant code frequencies within the 108 segments (a segment may be coded for more than one determinant, so the sum of the total numbers of coded segments is greater than 108). The left column lists each of our 5 determinates, and the right column lists the number of times a segment was designated to each corresponding code.

Determinant code	Total number of coded segments
Individual clinician authority	81
Communication, documentation, and administrative needs	40
Staffing and work schedule	37
Integration with existing information systems	26
Clinician self-identity and job satisfaction	28

Table 2 shows the frequency of co-occurrence of codes within interview segments. The most frequent and prominent code was individual clinician authority. This code also co-occurred most frequently with the other 4 determinants of implementation success within our interviews.

**Table 2:** Frequency of co-occurrence of determinant codes within interview segments. The numbers in each cell represent the number of times each pair of determinants was mentioned together within the same segment.

	Individual clinician authority	Communication, documentation, and administrative needs	Staffing and work schedule	Integration with existing information systems	Clinician self-identity and job satisfaction
Individual clinician authority	N/A^a^	42	32	21	24
Communication, documentation, and administrative needs	42	N/A	10	5	17
Staffing and work schedule	32	10	N/A	4	8
Integration with existing information systems	21	5	4	N/A	8
Clinician self-identity and job satisfaction	24	17	8	8	N/A

^a^N/A: not available.

In order to gain insight into the way the implementation and adoption process influences the use of the Dashboard (our second research objective), we evaluated the sentiment (positive or negative) associated with each pairing. Specifically, within each pairing, each of the 2 codes was identified and thematized as reflecting positively or negatively on that determinant. Therefore, in each pairing, both codes could be positive, both could be negative, or 1 negative and 1 positive.

Table 3 shows illustrative quote examples sorted by instances where both determinants reflected positive sentiments, both reflected negative sentiments or the scoring was mixed (negative/positive, positive/negative) between the 2 determinants. A more comprehensive table of relationships, code/determinant pairings, and illustrative quotes can be found in Multimedia Appendix 2.

**Table 3:** Relationships, code-determinant pairings, and illustrative quotes.

Relationships	Illustrative quotes of code-determinant pairings
Positive authority and positive other determinant	Individual clinician authoritywithcommunication, documentation, and administrative needs: “Providers are pretty receptive to hearing from us about dosing changes or drug interactions, or the questions that come up about these high-risk medications. They're familiar with a lot of our names because we're in touch with them about anti-coagulant questions in general…” [Pharmacist, Site B, ID004] Individual clinician authoritywithStaffing and work schedule: “...we have developed a system where usually, as long as we're fully staffed, one of the pharmacists is able to run the report for the day and kind of focus on that alone for the entire clinic day.... that helps me direct some of the more high-level alerts that we can take care of.” [Pharmacist, Site B, ID004]
Negative authority and negative other determinant	Authority withclinician self-identity and job satisfaction: ”It's just a massive report, where even though we can get through many alerts each day, it feels very insignificant sometimes because we're talking about thousands of alerts…it’s just a lot for one person to focus on.” [Pharmacist, Site B, ID004]
Positive authority and a negative other determinant	Individual clinician authorityandIntegration with existing information systems: “...when I reached out to them and said I really want the changes to go live, because this will optimize this program, I had to work with our IT group and ... select the top two that were a priority, out of a list of like 20 updates, just because they don't have the means to do it.” [Pharmacist, Site A, ID001]
Negative authority and a positive other determinant	No examples found

### Authority Within Interprofessional Collaboration and Communication

The EHR-based DOAC Dashboard itself has been a tool of empowerment for clinic staff, leading to streamlined operations that facilitate monitoring all DOAC-treated patients across a health system or managed by large physician groups at their hospitals. Interview participants indicated that they have the authority to routinely run various EHR reports monitoring DOAC-treated patients and the authority to create guidelines for the dashboard use and protocols regarding when staff should contact a physician about a patient’s medication errors, aiding the clinic in their communicative process.

Interviewees stated that having a trusting relationship between physicians and anticoagulation clinic staff had a positive effect on the success of the dashboard’s implementation. Physicians’ endorsement of the anticoagulation clinic had been instrumental in securing clinic resources associated with the implementation of the dashboard.

A lack of a trusting relationship with providers was cited as creating barriers to the successful implementation of the dashboard, as the anticoagulation clinic staff were reluctant to reach out to providers with questions. When trust was present, clinic staff could more easily coordinate medication adjustments with the physicians when they found a medication issue on the dashboard.

Various perceived barriers to the successful implementation of the DOAC Dashboard across departments and roles were mentioned related to a perceived lack of effective collaboration between interviewees working with the Dashboard and the prescribing counterparts. Examples include (1) slow responses to clinic staff’s inquiries, (2) prescribers’ resistance to contact from or input from nonphysicians, (3) prescribers’ formatting of notes in the EHR that trigger unnecessary alerts in the dashboard for the pharmacists or nurses to review, and (4) prescribers dismissing relevant information contained in dashboard alerts, requiring the pharmacist or nurse to follow-up on the alert.

### Authority Over Staffing and Scheduling Decisions

Overall, the interviewees expressed that the work with the EHR-based DOAC Dashboard was facilitated by the authority to split up the work between team members to balance the workload and to choose when to work on dashboard content. Working as a team to overcome the backlog and share the work was 1 strategy commonly cited. The chief barrier to adoption was the lack of prioritized and dedicated resources for dashboard work to address that backlog. Despite being an efficient and useful EHR tool, there were not enough resources (time and staffing) to fully leverage the power of the DOAC Dashboard. Because of the overwhelming workload and constrained staffing resources, clinic staff felt they could not responsibly expand the scope of their work without compromising quality.

### Individual Clinician Authority, Self-Identity, and Job Satisfaction

Interviewees stated that using the EHR-based DOAC Dashboard has been empowering, and thus has supported them in achieving meaningful work. One interviewee shared that since using the dashboard, they have been in contact more frequently with physicians to provide them with appropriate interventions for patient medication issues that they had been able to identify through their dashboard use. In addition to facilitating the collaboration between clinic staff and physicians, the dashboard’s “flagging” system has helped the clinic staff reduce unnecessary low-value patient calls and instead focus on reaching patients in greater need of interventions.

### Authority Regarding IT Integration

Several interviewees shared positive experiences working with IT staff while integrating the DOAC Dashboard at their clinic. The implementation was cited as positive and successful in instances where the clinic staff had the authority to work with their IT staff to adapt the dashboard to fit local needs, and IT staff could respond quickly and competently to their questions. However, for some, despite clinic staff authority, the lack of IT staff resources presented a barrier to dashboard integration with existing information systems. For instance, 1 interviewee shared that they were receiving alerts on medications that were not relevant, and despite engaging IT staff to resolve the issue, the problem has not been resolved due to limited IT staff resources in their health system.

## Discussion

### Principal Findings

Implementing technology in health care is both a common and complex endeavor. In this study, we have examined the ways in which the degree of clinical authority held by clinicians influences the success of implementation. Within the context of this study, individual clinician authority has referred to the power granted to clinicians to carry out role-related functions, as well as the autonomy that arises from their negotiations regarding the scope of power over other individuals and tasks. With regard to the DOAC Dashboard specifically, it includes medication-related authority, communication-related authority, workflow and staffing-related authority, and technology-related authority.

When implementing new EHR-based tools, addressing various domains of individual clinician authority is critical for success. Our data suggest that the DOAC Dashboard can empower the individual authority of pharmacists and nurses to ensure the safe use of DOACs (our first research question). Importantly, establishing and promoting individual clinician authority over how EHR-based tools are implemented and integrated into the workflow is associated with improved self-identity and job satisfaction while also promoting multidisciplinary collaboration.

Since the use of population health tools has become a necessary shift in anticoagulation stewardship, examining the relationship between the pairings of individual clinician authority and the other 4 determinants will help provide useful operational strategies and recommendations for potential users of the tool. The results suggest the ways in which the implementation and adoption process can facilitate or hinder the use of a DOAC Dashboard (our second research question).

### Individual Clinician Authority Is Needed to Facilitate Key Features of EHR-Based Tools

One characteristic of the EHR-based DOAC Dashboard is that it facilitates the ability to leverage multidisciplinary expertise for individual patients. By providing key information to an expert nurse or pharmacist, they can support an individual prescribing clinician on the nuances of evidence-based anticoagulant use. Our qualitative findings support the notion that a strong level of individual clinician authority to review DOAC prescriptions via the dashboard (reaching out to physicians concerning DOAC-treated patients’ dosing changes or drug interactions) has facilitated their communication and collaboration more broadly with physicians in their health system. While the communication between physicians and anticoagulation clinic staff (pharmacists or nurses) is affected by various contextual factors, most interviewees felt that the DOAC Dashboard has empowered their individual medication-related authority to oversee more DOAC-treated patients and reach out to more physicians to correct medication errors and answer questions about these high-risk medications.

Conversely, interviewees’ statements often reflected that a lack of individual authority was associated with negative themes regarding other determinants. For example, several interviewees felt powerless and frustrated when physicians ignored alerts associated with potentially dangerous drug interactions or did not follow the dashboard protocol. This is particularly important as clinical pharmacists and nurses do not have legal prescribing authority in Michigan, limiting their individual authority and making them reliant on physicians and other clinicians with prescribing authority. This demonstrated a critical barrier for any EHR tool design, which may be used by clinicians who do not have provider authority and require multidisciplinary collaboration.

EHR-based tools are often intended to improve efficiency and reduce staff workload. Several interviewees reported that the EHR-based DOAC Dashboard has allowed them to target patients who are most likely to require intervention, and thus has improved the flexibility and efficiency of their work schedule and facilitated better use of staffing resources. Nevertheless, interviewees also mentioned that the effective implementation of the DOAC Dashboard is determined by the existence of dedicated time and staff to work on the dashboard. This finding aligns with our previous research at the VA clinical sites [8], highlighting the importance of dedicated resources across health systems.

At the same time, issues of patient volume are important to address when implementing new EHR-based tools. One interviewee felt overwhelmed by the massive number of alerts generated using the DOAC Dashboard. Having insufficient workflow-related authority and guidance on how to prioritize and delegate these alerts led to unintended negative effects on the clinic staff’s job identity and satisfaction. As health systems grow and merge, the likelihood that EHR-based tools may present overwhelming numbers of patients for individual clinical staff to manage is a critical barrier to successful adoption.

As with any EHR-based tool, the availability and accessibility of IT staff are critical for successful implementation. Having the individual technology-related authority to engage the IT departments within health systems was cited as improving implementation by resolving technical issues with the dashboard and better integrating the new tool with their existing information systems. Additionally, the medication-related and workflow-related authority empowered through dashboard use to identify patients with the highest priority led to interviewees’ reports of meaningfulness of work and job satisfaction.

### Limitations

We acknowledge that our study is limited by a relatively small sample size. However, as noted above in the Methods section, this limited sample size comprises the overwhelming majority of users of this technology, and therefore is a valid and representative sample of the population. The value of this limited sample size is also bolstered by the fact that the data collected are a complement to the data from our previous research [25]. As in any qualitative interview, the interview questions have focused on the responses of respondents to the determinants identified in our previous research. We did not structure our interviews to specifically address each determinant of implementation success alongside individual clinician authority. Rather, our interview questions focused on general empowerment within their clinic and how the dashboard affects the empowerment of clinicians to ensure safer DOAC stewardship. Our analysis approach was also shaped by our previous research, as our coding scheme and the subsequent thematic analysis were developed based on our findings from 45 previous interviews (32 postimplementation from the VA sites and 13 preimplementation at non-VA sites).

### Differentiating Population Health Tools From CDS

Much of the IT literature has focused on the development and implementation of CDS within the EHR. CDS is designed to support individual clinicians in making individual decisions for individual patients. Population health tools, on the other hand, are designed to analyze data across a large population of patients and provide critical and actionable data to designated individuals who then support the primary clinicians. Nonetheless, the barriers identified for successful CDS implementation may overlap with those of population health tools [26]. Yet, a key distinction for population health tools that may not apply to CDS is the necessity to address issues of individual clinician authority. CDS tools typically target clinicians who have the authority to make changes to their own orders. Population health tools, as has been demonstrated in this work, may facilitate multidisciplinary collaboration but can be limited by the degree of individual clinician authority for whoever is using the EHR-based population health tool.

### Recommendations and Implications

The findings of this study have important implications for those who are tasked with implementing EHR-based tools within a clinical setting. Such implementation tasks are often challenging due to the lack of resources, and the inherent difficulties in implementing any change [27,28]. The results of this study suggest that creating a workplace culture that promotes individual clinician authority over their work contributes to the success of the implementation of an innovative intervention that relies heavily on interprofessional collaboration and communication.

Based on these findings, clinic managers and staff who plan to implement an EHR-based tool for population management, such as the DOAC Dashboard, should evaluate their site’s current culture of staff authority and make necessary changes to increase individual clinician authority as much as possible. Managers should consider eliciting feedback from staff regarding operational effectiveness to better understand their clinical staff’s needs. Additionally, staff can benefit from manager’s support of flexibility and autonomy in workflow, scheduling, and communication within and between departments as well as advocating for policies that enhance autonomy for their staff. It is well-established that implementing EHR-based tools involves more than providing the necessary software or programming. Our study provides evidence that increased individual clinician authority serves as a necessary antecedent to the effective implementation of EHR-based tools and very likely has positive effects on all other aspects of implementation.

### Conclusions

Individual clinician authority is a key determinant of successfully implementing EHR-based population management tools, such as medication dashboards for anticoagulation stewardship. We assert that positive individual authority granted to those responsible for the implementation of an EHR-based tool is interconnected with other determinants of success and has a positive effect on implementation.

While adjusting certain determinants of successful implementation (eg, staffing and IT staff resource availability) may not be possible, assuring clinic staff members have the necessary authority over their work is modifiable. Establishing a clinical service culture in which staff are involved in decisions related to the implementation of an EHR-based tool and its ongoing use is a foundational step in implementing a new program into a clinical setting.

Future research can further expand on specific, proactive strategies that may improve the implementation of EHR tools. In particular, this research suggests that expanding authority and autonomy may represent a low-cost strategy that can be accomplished without requiring constrained resources such as increased staffing levels. Rather, increased authority and autonomy may be an implementation strategy that allows existing resources to be used more effectively. If effective, such as strategy would be applicable well beyond this specific DOAC Dashboard application. Further research would enable a deeper understanding of the effects of this type of strategy.

## Appendix

Multimedia Appendix 1 Codes and definitions for qualitative content analysis.

Multimedia Appendix 2 More comprehensive table of relationshipsa, code-determinant pairings, and illustrative quotes.

## Abbreviations

CDS: clinical decision support
DOAC: direct oral anticoagulant
EHR: electronic health record
VA: Veterans Health Administration
