# Supplementary material for: Role of Individual Clinician Authority in the Implementation of Informatics Tools for Population-Based Medication Management: Qualitative Semistructured Interview Study
Source: JMIR Hum Factors. 2023 Oct 24;10:e49025. doi: 10.2196/49025 (PMC10630856; doi:10.2196/49025)
Supplement: Multimedia Appendix 1 [file humanfactors_v10i1e49025_app1.docx]

| **Code** | **Definition** |
| --- | --- |
| Individual clinician authority | The power granted to clinicians to carry out role-related functions, as well as the autonomy that arises from their negotiations regarding the scope of power over other individuals and tasks. Specifically, it includes:   - Medication-related authority: the ability to make evidence-based medication changes or provide recommendations for medication changes - Communication-related authority: the ability of clinicians effectively to communicate and collaborate with DOAC prescribers across various domains of knowledge and expertise - Workflow and staffing-related authority: the ability to personalize work schedule or workflow and influence decisions about staffing resources - Technology-related authority: the ability to address issues related to the integration of IT systems or the interaction with the dashboard to optimize the tool’s performance |
| Communication, documentation, and administrative needs | Communication (or lack thereof) with prescribing clinicians (e.g., physicians), and experiences with documentation of the large set of DOAC patients |
| Staffing and work schedule | Workload concerns about managing DOAC patients, impacts of other responsibilities on DOAC Dashboard time, or the amount of time available to focus on the dashboard |
| Integration with existing information systems | How well the dashboard integrates into the existing computer system, as well as relating to IT resources and the prioritization of implementation |
| Clinician self-identity and job satisfaction | Feelings about using the dashboard, and general job satisfaction |
