# Supplementary material for: Role of Individual Clinician Authority in the Implementation of Informatics Tools for Population-Based Medication Management: Qualitative Semistructured Interview Study
Source: JMIR Hum Factors. 2023 Oct 24;10:e49025. doi: 10.2196/49025 (PMC10630856; doi:10.2196/49025)
Supplement: Multimedia Appendix 2 [file humanfactors_v10i1e49025_app2.docx]

| **Code pair** | **Relationship^a^** | **Illustrative Quotes** |
| --- | --- | --- |
| Individual clinician authority / Communication, documentation, and administrative needs | Positive/ Positive | Providers are pretty receptive to hearing from us about dosing changes or drug interactions, or the questions that come up about these high-risk medications. They're familiar with a lot of our names because we're in touch with them about anticoagulant questions in general… (Pharmacist, Site B, ID004) |
|  | Negative/ Negative | "…there's a lot of physicians that free text their instructions and that's not the best way to do it… instead of selecting the computer frequencies they already have, they developed their own templates of these medications…” (Pharmacist, Site A, ID0001) |
|  | Positive/ Negative | “...we might get some resistance from another physician, which can be kind of understanding, because what we do is send them a telephone encounter and introduce that we are part of the DOAC dashboard, and then we go into why we do that, and then we talk about our findings, and if they're in agreement than we would write the prescription and do the teaching. Just you have a few doctors like, “Why are we doing that?” “that's [my] patient” (Nurse, Site D, ID005) |
|  | (-) / (+) | No instances found |
| Individual clinician authority / Staffing and work schedule | Positive/ Positive | "...we have developed a system where usually, as long as we're fully staffed, one of the pharmacists is able to run the report for the day and kind of focus on that alone for the entire clinic day.... There are thousands of patients on it so I like to filter the results into critical alerts, that helps me direct some of the more high-level alerts that we can take care of” (Pharmacist, Site B, ID004) |
|  | Negative/ Negative | “…if our patient transitions to a DOAC we get the pharmacist's help in that, they may tell us what to do… they determine all of that. We don't really have anything to do with dosing… We do the reports if we have time, it's not in our day-to-day everyday workload” (Nurse, Site B, ID006) |
|  | Positive/ Negative | "we provide this service and we have to provide that reliably for them...we're still working on catching up those alerts and making sure that we're able to manage these patients and the concern would be… if we opened it up to internal medicine and got thousands more patients that we wouldn't be able to get to those and there would be this false sense of security from the providers … and then we really aren't able to keep up with that workload." (Pharmacist, Site A, ID001) |
|  | (-) / (+) | No instances found |
| Individual clinician authority / Integration with existing information systems | Positive/ Positive | "...we work with the IT department. We added a comment section because we felt that we were going back into the whole chart. So right when the dashboard shows up like, your initial screen thing, we were able to put a little comment section so we could write up to like three lines so that we didn't have to go into the patient's chart, we could just write like a little comment on there. We added things to make it a little bit easier." (Nurse, Site D, ID002) |
|  | Negative/ Negative | "…If we want to snooze an alert, say the patient is on the wrong dose, but the physician particularly wants this patient on the wrong dose for some reason, once we snooze the alert, it snoozes all alerts from coming in. So, if the patient was put on an interacting med that alert wouldn't come through…" (Nurse, Site D, ID002) |
|  | Positive/ Negative | "...when I reached out to them and said I really want the changes to go live, because this will optimize this program, I had to work with our IT group and ... select the top two that were a priority, out of a list of like 20 updates, just because they don't have the means to do it" (Pharmacist, Site A, ID001) |
|  | (-) / (+) | No instances found |
| Individual clinician authority / Clinician self-Identity and job satisfaction | Positive/ Positive | "... just the fact that you get “Thank you’s” from these physicians, saying, “Oh, my gosh! I didn't realize that, yes please change the dose” you get the patients… they're like, “Oh, Thank you so much. I appreciate you checking on this.” So, it's just gratifying” (Nurse, Site D, ID002) |
|  | Negative/ Negative | " It's just a massive report, where even though we can get through many alerts each day, it feels very insignificant sometimes because we're talking about thousands of alerts. So, I think if we're able to ever get the report down to only critical alerts or a smaller number of alerts we will be able to feel like we're making a bigger outcome, it’s just a lot for one person to focus on.” (Pharmacist, Site B, ID004) |
|  | (+) / (-) | No instances found |

^a^Positive sentiments indicate the presence of the determinant, and negative sentiments indicate the absence of the determinant.
